# Firearm Injuries during Pregnancy in the USA

**DOI:** 10.3390/clinpract13040072

**Published:** 2023-07-09

**Authors:** Taylor Luster, Randall T. Loder

**Affiliations:** 1 Division of Student Affairs, Indiana University School of Medicine, Indianapolis, IN 46202, USA; 2 Department of Orthopaedic Surgery, Riley Children’s Hospital, Indiana University School of Medicine, Indianapolis, IN 46202, USA

**Keywords:** pregnancy, firearm injury, demographics, emergency department, mortality, fetus

## Abstract

Trauma during pregnancy is the leading cause of non-pregnancy-related maternal deaths, with some due to injuries from firearms. It was the purpose of this study to characterize the patterns and presentations of firearm-associated injuries in pregnant women using a national emergency department visit database. Data from the Inter-University Consortium for Political and Social Research Firearm Injury Surveillance Study 1993–2020 were utilized. The data include age, sex, race, type of firearm, perpetrator of injury, intent of injury (unintentional, assault, suicide, or law enforcement), anatomic location of the injury, incident locale, disposition from the emergency department (ED), and whether the patient was shot or not with the firearm. Of the 3.36 million ED visits over this time span for firearm injuries, 4410 were pregnant women. The mean age of the pregnant cohort was 23.6 years, with more Hispanic and fewer White women in the pregnant group compared to the non-pregnant cohort. Pregnant women were more likely to experience an injury involving the lower trunk and had a higher percentage of fatalities and hospital admissions compared to the non-pregnant cohort. Fetal demise occurred in at least 70% of cases. Nearly one half of the assaults (44%) occurred on Saturdays and Sundays. As the cause of these injuries is complex, prevention will require input from multiple sources, including health care providers, social agencies, government agencies, elected officials, and law enforcement.

## 1. Introduction

Trauma during pregnancy is the leading cause of non-pregnancy-related maternal deaths [1,2,3,4]. The management of trauma in pregnancy is challenging due to the increased difficulty of specific physiological demands due to hypoxia, hypotension, and hypovolemia [2,5,6] as well as a potentially viable second individual [7]. As high as 57% of traumatic maternal deaths may be due to homicide [8], with 22.7% of maternal deaths being from gunshot wounds [1].

Injuries due to firearms are a significant health burden in the United States, as the number of overall deaths attributed to firearms is equivalent to those from motor vehicle crashes and falls [9,10,11]. Firearm-associated injuries can result in significant trauma and death in pregnant women and is often associated with intimate partner violence [12,13,14,15,16]. Maternal intimate partner violence is associated with storing a loaded firearm in the home, and firearm injuries account for the greatest proportion of women’s mortality by an intimate partner in the US [13].

Despite these studies, there is little data regarding the demographics and associated injury patterns in pregnant women associated with firearm activity. Some studies of trauma during pregnancy only address maternal fatalities [1,4,14,15,16], while others investigate all causes of trauma without a particular focus on those due to firearms [1,2,3,4]. Details regarding the demographics and associated injury diagnoses/anatomic locations are either lacking or minimal. Other studies have discussed the care of pregnant trauma patients well, [2,5,6,7] but have not delineated the demographics of such injuries, and in particular those due to firearms. Studies of intimate partner violence in pregnant women involving firearms often discuss both fatal and non-fatal outcomes, but again, they do not give the injury details [12,13,17]. Thus, there is a large gap in the knowledge regarding the demographics and injury patterns associated with firearm use in pregnant women.

It was therefore the purpose of this study to characterize the patterns and presentations of firearm-associated injuries to pregnant women using a national emergency department visit database for both fatal and non-fatal outcomes. There is no national study like this to our knowledge. These results will be useful as baseline data for future studies and treatment strategies and may guide prevention methods. 

## 2. Materials and Methods

### 2.1. Data Source

Data from the Inter-University Consortium for Political and Social Research Firearm Injury Surveillance Study 1993–2020 (ICPSR 38574) (https://www.icpsr.umich.edu/web/NACJD/studies/38574) (Accessed on 18 December 2022) were used in this study. The ICPSR data are collected by the National Electronic Injury Surveillance System (NEISS). The NEISS, a branch of the US Consumer Product Safety Commission, collects data from a probability sample of hospitals in the United States and its territories that have at least six beds and an ED. The sample contains five strata, four based on size (the total number of emergency room visits reported by the hospital and are small, medium, large, and very large), and one stratum consisting of children’s hospitals. Hospital size (strata) is defined by the number of ED visits per year, which are small (0–16,830), medium (16,831–21,850), large (28,151–41,130), and very large (>41,130), and one encompassing children’s hospitals of all sizes. There are approximately 100 hospitals in the NEISS, and this number varies slightly from year to year. Patient information is collected daily from each NEISS hospital for every patient treated in the ED due to an injury associated with a consumer product. The ICPSR dataset consists of any patient seeking care in the ED for any firearm-related injury, regardless of activity involved during the injury (e.g., hunting, committing a crime, suicide, or assault), and whether the patient had been shot by the firearm or injured in some other way (e.g., a skull/face fracture from being pistol whipped, a clavicle fracture from a rifle recoil, etc.). From this weighted, stratified dataset, the actual number of ED visits (n) is used to obtain the estimated number (N) of ED visits for the entire US. Further details regarding the acquisition of the ICPSR/NEISS data and guidelines for use of such data can be accessed from their respective web sites (ICPSR—www.icpsr.umich.edu; NEISS—www.cpsc.gov/library/neiss.html) (Accessed on 12 December 2022). This study of publicly available de-identified data was considered exempt by our local Institutional Review Board.

The data from 1993 through 2020 were downloaded from the ICPSR website, as 2020 provided the most recent data when this study began in late 2022. There is typically a lag time of two to three years before the data are posted to the website. The data included age, sex, race, type of firearm, perpetrator of injury (e.g., self, stranger, etc.), intent of injury (unintentional, assault, suicide, or law enforcement), anatomic location of the injury, incident locale (home, street/highway, etc.), disposition from the ED, involvement with illicit drugs, the commitment of a crime or a fight/argument in the incident, and whether the patient was shot or not with the firearm. The anatomic location of the injury is defined as head/neck, upper trunk (above the navel), lower trunk (below the navel), upper extremity, and lower extremity. Race was classified as White, Black, Amerindian (Hispanic and Native American), and Asian [18]. 

Pregnancy was ascertained using the database column CMTX; this column is a description of the event where patient identification has been expunged. The database was searched for pregnancy using the FIND command for the terms preg, gesta, fetus, fetal, gravid, and expecting. The presence of a drive-by shooting was found by searching the database using the FIND command for the terms driveb, drive-by, drive-b, driveth, drive th, and drive-th. The gestational age of the pregnancy was also ascertained from the CMTX column when given and divided into trimesters, as defined by the American College of Obstetrics and Gynecology (https://www.acog.org/womens-health/faqs/how-your-fetus-grows-during-pregnancy) (Accessed on 24 January 2023): first trimester up to 13 weeks 6 days; second trimester 14 weeks to 27 weeks 6 days; and third trimester 28 weeks or more. We only included women who were 15–46 years of age in the analyses as this was the age range for the pregnant cases upon analysis of the dataset. This also reflects the prime age of reproductive years in women.

### 2.2. Statistical Analysis

When using weighted datasets, statistical analyses are usually performed to account for the weighted, stratified nature of the data, giving an estimated number of ED visits along with 95% confidence intervals (CI) of the estimate. However, when reviewing the very small number of 136 pregnancy encounters from the search, a weighted analysis national estimate was only performed for the entire group. Subsequent analyses simply used traditional non-weighted statistics, as for any typical cohort study. This is due to the fact that when the actual number of cases is <20, the weighted estimates become unstable and should be interpreted with caution. Differences between groups of categorical data were analyzed by the χ^2^ test (>2 × 2 analyses) or the Fishers exact test (2 × 2 analyses) test. Differences between continuous variables were analyzed using non-parametric methods (Mann–Whitney U test for 2 groups, and Kruskal–Wallis for 3 or more groups). Weighted analyses were performed with SUDAAN 11.0.01™ (RTI International, Research Triangle Park, North Carolina, USA 2013), while non-weighted analyses were performed with Systat 13.1 software (San Jose, CA, USA, 2009). For all analyses, a *p* < 0.05 was considered statistically significant.

## 3. Results

There were 12,703 women who were 15 to 46 years of age; 135 were pregnant and 12,568 were not. The descriptors mentioned pregnancy in 136 cases. An initial review of these 136 cases encountered a 4-year-old boy; all others were females between the ages of 15 and 46 years. The narrative comments for this particular boy stated that he was shot in the abdomen at an apartment complex. His pregnant mother and her boyfriend were shot and killed, and the child was admitted to the hospital. While this is a tragic case, it was excluded as the boy was not the pregnant patient seen in the ED. Over the 28-year period of 1993 through 2020, there were 111,796 actual ED visits for injuries due to firearms overall, resulting in an estimated 3,359,809 [2,956,755, 3,744,864] ED visits. Of these 3.36 million ED visits, the 135 actual pregnant patients correspond to an estimated 4410 [2832, 6818] pregnant patients injured due to firearm activity (4410/3,359,809—0.13% of all ED visits for firearm-associated injuries).

**Figure 1:**
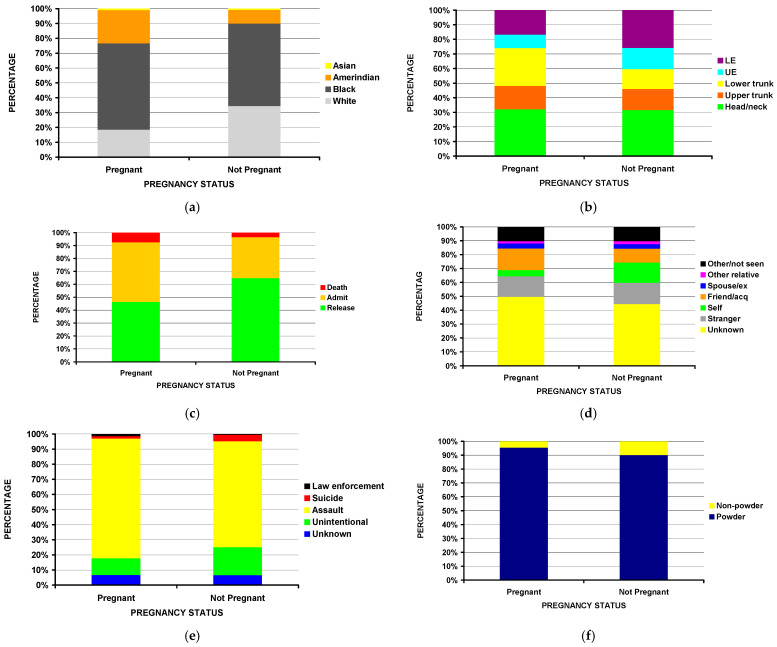
Differences in firearm injury patterns in pregnant women compared to non-pregnant women. (**a**) By race (*p* = 0.000004). (**b**) By anatomic location of injury (*p* = 0.00026). (**c**) By disposition from the ED (*p* = 0.00002). (**d**) By perpetrator (*p* = 0.022). (**e**) By injury intent (*p* = 0.028). (**f**) By firearm type (*p* = 0.04).

The mean age of the pregnant cohort was 3.3 years less than the non-pregnant cohort (23.6 vs. 26.9 years, *p* = 0.00009) (Table 1). There was a marked difference by race (Figure 1a), with a higher proportion of Amerindian women and a lower proportion of White women in the pregnant group (*p* = 0.00004). The anatomic area of injury also differed (*p* = 0.00026), with a higher proportion of lower trunk injuries in the pregnant cohort (26.0% vs. 13.6%) and a lower proportion of lower extremity (16.8% vs. 25.9%) and upper extremity injuries (9.2% vs. 14.6%) in the non-pregnant cohort (Figure 1b). The pregnant cohort had a higher percentage of fatalities (7.5% vs. 3.5%) and hospital admissions (46.3% vs. 31.7%) compared to the non-pregnant cohort (*p* = 0.00002) (Figure 1c). Self-inflicted injuries were less frequent in the pregnant cohort when compared to the non-pregnant cohort (*p* = 0.022) (Figure 1d). This aligns with the findings that the intent of the injury was more commonly an assault in the pregnant cohort compared to the non-pregnant cohort (79.3% vs. 70.1%) (*p* = 0.028) (Figure 1e). While most of the injuries involved powder firearms, the percentage of powder firearm injuries in the pregnant cohort was greater (95.6% vs. 90.1%) (*p* = 0.04) (Figure 1f).

### 3.1. Within the Pregnancy Cohort

The gestational age of the pregnancy was known in 92 of the 136 patients. There were 20 injured women (22%) in the first trimester, 53 (58%) in the second trimester, and 19 (20%) in the third trimester. There were no differences by trimester for the age of the patient, disposition from the ED, or race (*p* = 0.53, 0.92, and 0.28, respectively). Fetal outcome was mentioned in twelve of the patients; the fetus died in nine cases, there was an unknown outcome in two cases, and the fetus was alive in one case (at the time of discharge from the ED). Automobiles were involved in at least nineteen of the cases; nine involved the patient sitting in the car or a carjacking; seven were when the patient was riding/driving in the car; one was when the patient actually lived in the car; and two were miscellaneous. There was a non-uniform distribution of all assaults on pregnant women by weekday, with 44% occurring on Saturdays and Sundays (60 of 135, *p* = 0.007).

We next analyzed those within the pregnancy cohort by being shot or not shot (Table 2). There were 93 patients shot (73%), and 37 (27%) were not shot. The only significant differences between the groups were for the anatomic location of the injury, injury diagnosis, and disposition from the ED. All the fatalities occurred in those who were shot (*p* = 0.00003). Injuries to the head/neck were much higher in the not-shot group (53% vs. 25%), as were lower trunk injuries (38% vs. 22%) (*p* = 0.003). By definition of not being shot, the not-shot group had no punctures as the diagnosis and a greater proportion of internal organ injuries (14% vs. 7%) and contusions/abrasions (24% vs. 4%) (*p* = 0.000002).

### 3.2. Case Examples

*Intentional shootings*

A 20-year-old pregnant patient was driving a car when she was shot at multiple times in a drive-by shooting. She hit a tree and sustained multiple gunshot wounds to the chest and abdomen with fetal demise.

A 16-year-old, who was 6 weeks pregnant, was dropped off at the ED by a friend for a gunshot wound to the abdomen.

A 25-year-old, who was 8 ½ months pregnant, was shot in the head and chest by her husband while at the court house. The fetus was delivered after the patient died.

A 33-year-old, who was 38 weeks pregnant, sustained a self-inflicted gunshot wound to the head. Cardiac arrest ensued and a peri-mortem cesarean section was performed.

*Accidental shootings*

A 21-year-old pregnant patient was cleaning her gun and it went off, resulting in a gunshot wound to the abdomen.

A 23-year-old, who was 6 weeks pregnant, sustained an accidental gunshot wound to the chest when she removed a gun from storage; when it went off, the bullet struck her, resulting in a chest wound and liver laceration.

A 17-year-old pregnant patient was accidentally shot in the chest when her husband unloaded his 380-caliber gun, resulting in her death. The husband only sustained a hand injury.

*Injuries besides shootings*

A 19-year-old, who was 2 months pregnant, was assaulted and pistol whipped to her head by her significant other. She was hit in the face and abdomen, resulting in a scalp hematoma and uterine pain.

A 36-year-old, who was 5 months pregnant, was held with a gun to her head while being sexually assaulted, including both vaginal and rectal rape.

A 17-year-old pregnant patient was punched in the head and pistol whipped by her 29-year-old boyfriend. This occurred in a parking lot, and then her head was slammed against the concrete, resulting in a loss of consciousness and concussion.

A 16-year-old pregnant patient was beaten by her boyfriend using his fists and the butt of the gun to the head, abdomen, and back, resulting in microscopic hematuria.

## 4. Discussion

Injuries associated with firearms presented to US’ EDs accounted for 0.13% of all firearm-associated ED injuries from 1993 to 2020. Within women aged 15 through 46 years, the fatality rate was 2.1 times greater in the pregnant cohort compared to the non-pregnant cohort; 81% of cases were women of color, and over one-half occurred in the second trimester of pregnancy. Fetal demise occurred in at least 7%. The weekend days of Saturday and Sunday are especially risky for pregnant women. 

When excluding non-pregnancy-associated conditions, the leading cause of death during pregnancy is trauma [1,2,3,4,7], which can be either intentional (intimate partner violence, assault, etc.) or unintentional (motor vehicle crashes, falls, etc.). In a small series from New Orleans of 25 pregnant women sustaining trauma [19], penetrating trauma (stabbings, gunshot wounds, etc.) occurred in 25% of cases. In a study from San Diego [2], violent assaults accounted for 12% of the 114 injured pregnant patients; the mechanism of the assault was not given. In a study of 95 maternal deaths from Cook County, Illinois [1], trauma was the cause in 44 patients; of these 44, 57% were homicides. The mechanism of injury in these 44 deaths was a gunshot wound in 20.5%, motor vehicle crashes in 20.5%, stabbings in 13.6%, strangulation in 13.6%, blunt head injuries in 9.1%, burns in 6.8%, and others in 7.1%. The fatality rate in this study of injuries associated with firearms was 7.5%. This is lower than the above studies because both survivals and deaths were included. It is also likely lower due to the fact that those dead at the scene were taken directly to the morgue, bypassing the ED, resulting in a lower number of such cases. Despite these limitations, the fatality rate associated with firearms was 2.1 times greater in the pregnant cohort compared to the non-pregnant cohort.

In a Maryland study of deaths during pregnancy and the first postpartum year, the leading cause of death was homicide [14]. Black women, those younger than 25 years, and unmarried women were at the highest risk of homicide; firearms were the most frequent method of murder (61.8%). They also noted that homicides during pregnancy were most prevalent (49%) during the first trimester and least prevalent (14%) during the third trimester. This is different from our study, where 22% were in the first, 58% in the second, and 20% in the third trimester of pregnancy, realizing that our study looked at all firearm-associated injuries, not just homicides. Intimate partner homicides increase with both alcohol consumption and firearm ownership [17]. Additionally, highly restrictive firearm carry laws also increase the incidence of intimate partner homicide [17], suggesting that more aggressive firearm legislation might actually increase intimate partner homicide. It seems contradictory that intimate partner homicide increases with increased firearm ownership while at the same time highly restrictive firearm carry laws also increase the incidence of intimate partner homicide, and that was clearly recognized by Roberts [17] with the statement, “The finding that carry laws have no significant effect on IPF homicide goes beyond my ability to conjecture, except to suggest that firearm accessibility related to intimate lethal violence in the United States is not affected by carry laws”.

Maternal intimate partner violence has been associated with storing a loaded firearm in the home, and in one US study [13], firearm injuries accounted for the greatest proportion of women’s mortality by an intimate partner. In a study from North Carolina [12], firearm use was reported in 23.1% of cases (101 of 406) of non-fatal intimate partner violence. In this study, only 3.7% of the pregnancy-associated firearm injuries were due to intimate partner violence. However, four of the five that were caused by a spouse or ex-spouse were intentional, with one being accidental. This accidental case involved a 17-year-old shot in the chest when her husband was unloading his 380-caliber gun, resulting in her death. By medical specialty, obstetricians and gynecologists report the highest level of new patient intimate partner violence screening, but only at 17% [20]. If intimate partner violence screening is expanded, greater numbers of intentional firearm injuries due to intimate partner violence could be potentially prevented.

The lower number of first-trimester pregnancies may indicate that the women did not yet know they were pregnant. Late pregnancy awareness (≥7 weeks gestation) occurs in 23% of women [21]; it is more common in poor non-White women who are younger and have had less education [22]. The high proportion of Black and Amerindian women (80.6%) in this study supports the lower number of first-trimester pregnancies observed in this study. The lower number of third-trimester pregnancy trauma victims may stem from a decrease in the overall level of activity during this stage of pregnancy [23,24,25]. Also, women of color more often experience pre-term labor and give birth in the second trimester, and as women of color constituted a large proportion in this study, this may also be an explanation for the fewer numbers in the third trimester [26]. It should usually be apparent to an ED provider that a woman is in her third trimester after a simple cursory physical examination. Finally, the database only uses the ED notes and may not reflect an in-depth medical record search for exact dating; some patients may be off by dates, which are then corrected in a detailed note by the obstetrician. Thus, the lower third trimester number is likely accurate. It must be remembered that the pregnancy date was likely given by the patient to the treating physicians in the ED. Knowing the trimester is important, as if the mother is near death and in the third trimester, a cesarean section may aid in maternal resuscitation and may also save the fetus if conducted within minutes after the mother’s vital signs have ceased and if vital signs are still present in the fetus after maternal cardiac arrest [7].

Trauma to a pregnant mother is more fatal to the fetus than the mother [7]. In a 1989 study from New Orleans [19], 8% of the mothers and 28% of the fetuses died after a traumatic event to the mother/fetus couple. Shah et al. [2] found that morbidity and mortality for the mother from trauma to the mother were not increased during pregnancy. However, maternal death, a high maternal Injury Severity Score (mean of 34.6—fetal loss; 7.4—no loss), serious abdominal injury, and hemorrhagic shock were risk factors for fetal death [2]. An increased risk of fetal death has also been noted in pregnant-sustaining orthopedic injuries [3]. Unfortunately, this dataset focuses solely on the mother and not on the fetus, as statistics regarding fetal outcomes are not uniformly recorded in this NEISS database. However, the number of fetal deaths was sobering when mentioned; it was 75%. This is due to the fact that many of these fetal deaths occurred in first and second-trimester pregnancies and, as such, would not be viable even with emergent deliveries. Interestingly, the lower trunk was injured nearly twice as often in the pregnant cohort compared to the non-pregnant cohort (26.0% vs. 13.6%). It could be surmised that the perpetrator especially intended to harm the unborn child (as well as the mother). However, to answer this question, information from the perpetrators would need to be known, and it is not available in the NEISS dataset. 

When injuries due to firearms are mentioned, they are initially associated with the person being shot. However, firearms can cause other injuries, such as head injuries from being pistol whipped or shoulder injuries from rifle recoils. In this study, 73% of the patients were shot. Several of the case examples demonstrate the types of injuries that result from not being shot (sexual assault at gun point, pistol whipping, etc.). This is important and new information for health care providers to know, alerting them to the issue of firearm violence without the patient being shot and suggesting that they should keep this in mind when evaluating pregnant trauma patients.

Weekends (Saturdays and Sundays) were especially risky for pregnant women, as 44% of the cases occurred on those days. This agrees with Saturday/Sunday being the peak time for firearm injuries in general [27]. This is likely due to the fact that on Saturdays and Sundays, patients and assault perpetrators have more free time and thus, a higher chance of such events occurring. Finally, hospital size can be used as a proxy for rural vs. urban locations, i.e., small hospitals are more likely located in rural areas and the large/very large hospitals in urban areas. With this background, the pregnant patients were less commonly seen in smaller hospitals compared to the non-pregnant cohort. We surmise that this is due to the fact that patients with firearm injuries seen in smaller (rural) hospitals are more likely due to rural-associated activities, such as hunting [28], and that those injuries due to assaults and firearm violence in larger hospitals are due to a higher rate of violence, including firearms, in urban locations. Also, it is very likely that a woman, knowing that she was pregnant, would go hunting; however, this is simply conjecture.

Can these results be used to guide prevention strategies? As 79% of the pregnant cohort were assaults, knowing the “cause/reason” for the assault would be beneficial from a preventative standpoint. However, assaults may be due to many different factors—random events, robberies, sexual assaults, or intimate partner violence, to name a few. It would be difficult to create prevention strategies for such a mixed group of reasons. The injuries were unintentional in 11%, and perhaps education regarding safe handling of firearms would be helpful here. However, the literature is disparate on the effectiveness of firearm safety training [29,30]. Proper firearm storage education would be helpful for the non-assault injuries [31]. However, implementation of secure firearm storage may require addressing fears of home intruders and increasing awareness of the risks associated with household firearm access [31]. This might be very difficult to achieve if the patient lives in areas of increased gun violence, as it is known that gun violence typically clusters in certain areas [32,33]. Another prevention strategy would be for health care practitioners of pregnant women to recommend no firearms in the home, as well as avoid potentially dangerous areas of the cities. However, avoiding dangerous areas may be impossible due to the area of residence where the women live. An assessment of potential firearm exposure to pregnant women could be enhanced by routing questioning of access to firearms in the household during routine prenatal visits, as has been proposed for pediatricians, as well as general firearm safety for families [34,35,36,37,38,39]. Passive actions, such as increasing the green space in firearm-injury-prone areas [40] and the demolition of abandoned buildings [41] are other possibilities. Online maps showing locations for voluntary, temporary firearm storage (especially useful for suicide victims) [42] and social media applications [43] are further possibilities. Community-based programs, such as gun buy-backs and other community-based prevention programs may be helpful, but they need to be deployed in culturally sensitive and trauma-informed manners, address social determinants of health, be appropriately funded, and include proper personnel training [44].

What is the health care providers’ role in firearm injury prevention? It cannot be better stated than by Abdallah and Kaufman [45], quoting from their abstract: 

“Physicians can intervene through screening, counseling, community engagement, and advocacy, and can mobilize the health care systems they serve to engage with injury prevention. Physicians also play a key role in expanding the knowledge base on firearm injury through much-needed research on the epidemiology, context, and outcomes of firearm injury. When we treat firearm injury as a disease, we can develop and implement interventions from the clinic to the statehouse that can curb profound harms. This work and these opportunities belong not only to emergency physicians and trauma surgeons, but to all fields that evaluate and assess patients over the life course”.

This will require a multidisciplinary approach, involving the fields of medicine, social work, law enforcement, government institutions, and elected officials/politicians [46]. In the meantime, health care providers can use the 5A’s approach [47] when discussing firearms with patients; these 5As are ask, advise, assess, assist, and arrange. While this sounds very nice theoretically, the pressures on clinicians in daily practice (more patients, inefficient electronic medical records, etc.) may make this difficult to achieve from a practical viewpoint. Legally, physicians have the right to discuss firearm safety issues with their patients [48] which has been previously challenged. 

The limitations of this study need to be acknowledged. One is the accuracy of the NEISS data. However, previous studies [49,50], including those involving firearms, have demonstrated over 90% accuracy of NEISS data. Second, this study only analyzes patients seen in EDs; thus, those visiting urgent care centers or other outpatient clinics are not captured in these data. However, we suspect that any serious firearm injury would be seen in an ED. Third, regional-specific analyses could not be conducted due to the de-identified nature of each hospital in the NEISS sample. It would be very interesting to study differences by region [51], especially regions with stricter gun control laws compared to others, but unfortunately, that is not possible, due to the de-identified status of each NEISS hospital. Fourthly, it is possible that some pregnant patients as well as those involved in drive-by shootings did not share that they were pregnant with the ED personnel and thus were not captured by the NEISS. As this is an ED-focused database, we have no information on the length of stay for those admitted to the hospital and accurate fetal outcome information. Finally, it is likely that the number of deaths is less than what actually occurred for both the pregnant and non-pregnant cohorts—those pronounced dead at the scene were likely not taken to the ED but rather directly to the morgue; that number is, however, unknown.

A major strength of this study is that it provides a national picture of injuries associated with firearms in pregnant women spanning a quarter of a century. It encompasses both rural and urban areas and all ethnic groups. These data will also be helpful in analyzing any changes in prevalence or demographics with any future firearm legislation, either for or against gun control. There has been a recent call for [52] and surge in firearm injury research, which in the past was less than optimal [53], and in fact, it was not until the late 20th century that firearm violence was considered a public health problem [54]. This study adds another small but important piece to the literature involving vulnerable members of society—pregnant women and unborn children simultaneously.

## 5. Conclusions

This study investigated injuries to pregnant women due to firearms using a national US ED database spanning 27 years. This is the first study of its kind, with many new findings. While these injuries are rare compared to the overall percentage of firearm injuries in the US, our results found that both the mother and fetus are at significant risk of death. Various prevention strategies, such as proper firearm storage, health care providers counseling pregnant women regarding the potential for serious injury due to firearms, improving the infrastructure of communities, and community-based programs are proposed based on our data. As the cause of these injuries is complex and multifactorial, prevention of these injuries will require input from multiple sources, including health care providers, social agencies, government agencies, elected officials, and law enforcement.

## Figures and Tables

**Table 1:** Demographics and injury patterns due to firearm activity for females aged 15 through 46 years old by pregnancy status *.

	Pregnant		Not Pregnant		*p* Value
	135		12,568		-
**Age (mean ± 1 sd)**	23.6 ± 5.8		26.9 ± 8.6		0.00009
**Race**	**n**	**%**	**n**	**%**	
**White**	19	18.4	3127	34.4	0.000004
**Black**	60	58.3	5063	55.6	
**Amerindian**	23	22.3	832	9.1	
**Asian**	1	1.0	77	0.8	
**Anatomic area injured**					
**Head/neck**	42	32.1	3827	31.5	0.00026
**Upper trunk**	21	16.0	1760	14.5	
**Lower trunk**	34	26.0	1645	13.6	
**Upper extremity**	12	9.2	1769	14.6	
**Lower extremity**	22	16.8	3138	25.9	
**Major diagnosis**					
**Puncture**	31	23.0	2398	20.4	0.46
**Laceration**	7	5.2	1278	10.9	
**Foreign body**	7	5.2	812	6.9	
**Fracture**	8	5.9	911	7.7	
**Internal organ injury**	12	8.9	872	7.4	
**Contusion/abrasion**	13	9.6	1113	9.5	
**Strain/sprain**	1	0.7	149	1.3	
**Concussion**	2	1.5	74	0.6	
**Hematoma**	1	0.7	53	0.5	
**Other/not stated**	53	39.3	4117	35.0	
**Firearm type**					
**Powder**	129	95.6	10,804	90.1	0.04
**Non-powder**	6	4.4	1183	9.9	
**Shot**					
**Yes**	98	72.6	8763	73.1	0.92
**No**	37	27.4	3224	26.9	
**Drive-by shooting**					
**Yes**	5	3.7	282	2.4	0.25
**No**	130	96.3	11,705	97.6	
**Intimate partner violence**					
**Yes**	5	3.7	363	3.0	0.61
**No**	130	96.3	11,624	07.0	
**Sexual assault**					
**Yes**	2	1.5	496	4.1	0.18
**No**	133	98.5	11,491	95.9	
**Disposition from the ED**					
**Release**	62	46.3	7667	64.7	0.00002
**Admit**	62	46.3	3757	31.7	
**Death**	10	7.5	417	3.5	
**Who caused the injury**					
**Unknown**	67	49.6	5323	44.4	0.022
**Stranger**	20	14.8	1829	15.3	
**Self**	6	4.4	1756	14.6	
**Friend/acquaintance**	21	15.6	1201	10.0	
**Spouse/ex**	5	3.7	391	3.3	
**Other relative**	2	1.5	252	2.1	
**Other/not seen**	14	10.4	1235	10.3	
**Intent of the injury**					
**Unknown**	9	6.7	791	6.6	0.028
**Unintentional**	15	11.1	2217	18.5	
**Assault**	107	79.3	8402	70.1	
**Suicide**	2	1.5	520	4.3	
**Law enforcement**	2	1.5	57	0.5	
**Incident locale**					
**Unknown**	53	39.3	4920	41.0	0.90
**Home/apartment**	36	26.7	3164	26.4	
**School/recreation**	2	1.5	330	2.8	
**Street/highway**	25	18.5	1907	15.9	
**Other property**	19	14.1	1653	13.8	
**Farm**	0	0.0	13	0.1	
**Marital status**					
**Not stated**	54	46.6	4568	44.2	0.094
**Married**	49	42.2	3999	38.7	
**Never married**	8	6.9	1148	11.1	
**Divorced**	0	0.0	364	3.5	
**Separated**	5	4.3	258	2.5	
**Hospital size**					
**Small**	1	0.7	740	6.2	0.00006
**Medium**	10	7.4	1239	10.3	
**Large**	40	29.6	1918	16.0	
**Very large**	82	60.7	7663	63.9	
**Childrens**	2	1.5	427	3.6	
**Argument**					
**Unknown**	94	69.6	7656	63.9	0.27
**Yes**	12	8.9	1000	8.3	
**No**	29	21.5	3331	27.8	
**Crime**					
**Unknown**	86	63.7	6963	58.1	0.33
**Yes**	22	16.3	1989	16.6	
**No**	27	20.0	3035	25.3	
**Drugs**					
**Unknown**	90	66.7	8090	67.5	0.16
**Yes**	10	7.4	496	4.1	
**No**	35	25.9	3401	28.4	
**Fight**					
**Unknown**	82	60.7	7182	59.9	0.14
**Yes**	21	15.6	1312	10.9	
**No**	32	23.7	3525	29.4	

* Not all variables have data for every patient; thus, the sum of a particular variable will not always equal the overall sum.

**Table 2:** Demographics and injury patterns in the pregnant cohort by being shot or not shot *.

	Shot		Not Shot		*p* Value
	98		37		-
**Age (mean ± 1 sd)**	23.7 ± 5.8		23.4 ± 5.9		0.63
**Race**	**n**	**%**	**n**	**%**	
**White**	14	19	5	17	0.92
**Black**	42	58	18	62	
**Amerindian**	17	23	6	21	
**Anatomic area injured**					
**Head/neck**	24	25	0	0	0.0003
**Upper trunk**	19	20	2	6	
**Lower trunk**	21	22	13	38	
**Upper extremity**	12	12	0	0	
**Lower extremity**	21	22	1	3	
**Major diagnosis**					
**Puncture**	31	32	0	0	0.000002
**Laceration**	3	3	4	11	
**Foreign body**	7	7	0	0	
**Fracture**	8	8	0	0	
**Internal organ injury**	7	7	5	14	
**Contusion/abrasion**	4	4	9	24	
**Strain/sprain**	0	0	1	3	
**Concussion**	0	0	2	5	
**Hematoma**	0	0	1	3	
**Other/NS**	38	39	15	41	
**Firearm type**					
**Powder**	93	95	36	97	1.00
**Non-powder**	5	5	1	3	
**Drive-by shooting**					
**Yes**	5	5	0	0	0.32
**No**	93	95	37	100	
**Sexual assault**					
**Yes**	0	0	2	5	0.18
**No**	98	100	35	95	
**Disposition from the ED**					
**Release**	32	33	30	81	0.000003
**Admit**	55	57	7	19	
**Death**	10	10	0	0	
**Pregnancy trimester**					
**1st**	16	23	4	18	0.80
**2nd**	39	56	14	64	
**3rd**	15	21	4	18	
**Who caused the injury**					
**Unknown**	55	56	12	32	0.05
**Stranger**	11	11	9	24	
**Self**	6	6	0	0	
**Friend/acq**	12	12	9	24	
**Spouse/ex**	3	3	2	5	
**Other relative**	2	2	0	0	
**Other/not seen**	9	9	5	14	
**Intent of the injury**					
**Unknown**	9	9	0	0	0.13
**Unintentional**	13	13	2	5	
**Assault**	73	74	34	92	
**Suicide**	2	2	0	0	
**Law enforcement**	1	1	1	3	
**Incident locale**					
**Unknown**	38	39	15	41	0.92
**Home/apartment**	27	28	9	24	
**School/recreation**	1	1	1	3	
**Street/highway**	19	19	6	16	
**Other property**	13	13	6	16	
**Marital status**					
**Not stated**	39	46	15	47	0.30
**Married**	38	45	11	34	
**Never married**	5	6	0	0	
**Divorced**	0	0	3	9	
**Separated**	2	2	3	9	
**Hospital size**					
**Small**	1	1	0	0	0.18
**Medium**	7	7	3	8	
**Large**	28	29	12	32	
**Very large**	62	63	20	54	
**Childrens**	0	0	2	5	
**Argument**					
**Unknown**	64	65	30	81	0.21
**Yes**	10	10	2	5	
**No**	24	24	5	14	
**Crime**					
**Unknown**	64	65	30	81	0.21
**Yes**	10	10	2	5	
**No**	24	24	5	14	
**Drugs**					
**Unknown**	66	67	24	38	0.96
**Yes**	7	7	3	4	
**No**	25	26	37	58	
**Fight**					
**Unknown**	64	65	18	49	0.14
**Yes**	8	8	13	35	
**No**	26	27	6	16	

* Not all variables have data for every patient; thus, the sum of a particular variable will not always equal the overall sum.

## Data Availability

These data are freely to anyone online at the Inter-University Consortium for Political and Social Research Firearm Injury Surveillance Study 1993–2020 (ICPSR 38574) (https://www.icpsr.umich.edu/web/NACJD/studies/38574, accessed on 18 December 2022).
